# The Association of Formula Protein Content and Growth in Early Infancy: A Systematic Review and Meta-Analysis

**DOI:** 10.3390/nu14112255

**Published:** 2022-05-28

**Authors:** Qiqi Ren, Kaifeng Li, Han Sun, Chengdong Zheng, Yalin Zhou, Ying Lyu, Wanyun Ye, Hanxu Shi, Wei Zhang, Yajun Xu, Shilong Jiang

**Affiliations:** 1 Innovation Center, Nutrition and Metabolism Research Division, Heilongjiang Feihe Dairy Co., Ltd., C-16, 10A Jiuxianqiao Rd., Chaoyang, Beijing 100015, China; rqqrenqiqi@163.com (Q.R.); likaifeng@feihe.com (K.L.); sunhan@feihe.com (H.S.); zhengchengdong@feihe.com (C.Z.); zhangwei1@feihe.com (W.Z.); 2 PKUHSC-China Feihe Joint Research Institute of Nutrition and Healthy Lifespan Development, Xueyuan Road 38, Haidian, Beijing 100083, China; 1911110168@bjmu.edu.cn (Y.Z.); lybjmu@126.com (Y.L.); yewanyun_vera@bjmu.edu.cn (W.Y.); 1610306136@bjmu.edu.cn (H.S.); 3 Department of Nutrition and Food Hygiene, School of Public Health, Peking University, Xueyuan Road 38, Haidian, Beijing 100083, China; 4 Beijing Key Laboratory of Toxicological Research and Risk Assessment for Food Safety, Peking University, Xueyuan Road 38, Haidian, Beijing 100083, China

**Keywords:** infant formula, protein/energy ratio, breastfed, weight gain, height gain

## Abstract

This systematic review aimed to examine differences in growth outcomes between breastfed infants and infants fed with formula with different protein/energy ratios during the first six months of life. We conducted a systematic review in the PubMed, Web of Science, and Springer databases. Twenty clinical trials qualified for inclusion. We extracted data about the growth outcomes of infants who were exclusive breastfed or exclusively infant formula fed in the first six months and used a meta-analysis to pool the finding data. We categorized study formulas into four groups according to their protein content: <1.8, 1.8–2.0, 2.1–2.2, and >2.2 g/100 kcal. In the first month of life, growth was not different between formula- and breastfed infants. During 2–3 months of life, growth was faster in infants who consumed formulas with protein contents higher than 2.0 g/100 kcal. After 3 months, formula-fed infants grew faster than breastfed infants. Our meta-analysis indicated that the growth outcomes of infants fed with infant formula with a relatively low protein/energy ratios, compared with that a relatively high protein/energy ratio, were close to those of breastfed infants.

## 1. Introduction

Human milk is presumably the ideal source of nutrition for infants. Briefly, human milk provides infants with appropriate amounts of (1) water; (2) building blocks of the body, such as amino acids and minerals; (3) metabolic fuels, such as lactose and triglycerides, (4) coenzymes and protein cofactors, such as vitamins and minerals required for enzymatic activities and protein functions; (5) bioactive molecules, such as miRNA and metabolites that regulate biochemical pathways; (6) immunoproteins, such as immunoglobulins and lactoferrin, that grant infants protection against infections; (7) oligosaccharides that nourish infant gut microbiota; etc. [1].

Because human milk contains a comprehensive profile of nutrients, the World Health Organization (WHO) defines exclusive breastfeeding as providing human milk as the sole source of nutrition—with no additional fluid or other food provided—within the first 6 months of life. Continued breastfeeding beyond 6 months with the introduction of complementary foods is also encouraged [2]. Nevertheless, the global rate of exclusive breastfeeding was only 38% in 1995 [3]. High-income countries generally have higher rates of exclusive breastfeeding than low-income countries [4]. Factors such as insufficient maternal leave, lack of training from hospitals, maternal diseases and psychological problems, etc. discourage the practice of exclusive breastfeeding [5].

Not exclusively breastfeeding is associated with short- and long-term health issues. Overall, inadequate breastfeeding leads to substantial morbidity and approximately 600,000 child deaths and an additional 100,000 maternal deaths annually [6]. During lactation, because infant formula lacks protective compounds in natural human milk, the rates of otitis media and diarrhea in nonbreastfed infants are 8% and 17% higher, respectively, than those in exclusively breastfed infants [7]. In the long run, breastfed infants have higher scores on intelligence tests and lower rates of chronic diseases than formula-fed infants [8]. In fact, the concept of developmental origins of health and diseases (DOHaD) suggests that early infancy is a window of susception when inappropriate nutrition is related to increased risks of chronic diseases later in life [9]. Particularly, accelerated postnatal weight gain has been associated with overweightness and disorders in glucose homeostasis and appetite control in adulthood [10,11]. Presumably, healthy, exclusively breastfed infants exhibit the ideal growth trajectory of early postnatal growth [12]. In feeding studies, formula-fed infants usually gained weight faster than breastfed infants [13]. 

Inappropriate macronutrient composition is one of the possible reasons for the higher growth velocity in formula-fed infants. The protein content in infant formula displays marked differences from that in human milk. First, to compensate the different amino acid profiles and lower protein quality, infant formulas provide higher protein content than human milk [14]. Additionally, the protein concentration decreases gradually throughout lactation, whereas the protein content in infant formulas remains the same [15]. Accordingly, infant formulas are designed to meet the protein requirements during the earliest postnatal phase, when the human milk protein content is the highest [15,16]. Consequently, infant formulas provide excessive protein for infants during most postnatal periods.

The relationship between formula protein content and faster growth in formula-fed infants has been investigated in both clinical trials and meta-analyses. Several clinical trials have demonstrated that infants fed with formula with lower protein content grew slower and more closely to breastfed infants than infants fed with formula with higher protein content. These clinical trials were identified and listed in the systematic reviews by Abrams et al. and Patro-Gołąb et al., which, to the best of our knowledge, are the only two systematic reviews currently available that summarized the works on infant formula protein contents and postnatal growth [16,17]. Unfortunately, neither of these two studies compiled data from different studies together, possibly because of the inhomogeneity in protein content, duration of feeding, infant age, etc. among different studies. In our initial exploration, we found that, by compiling data from different clinical trials together, it was possible to establish the dose-dependent effects of formula protein content on infant growth and to demonstrate the effects of formula feeding on infant body growth during different postnatal stages when human milk protein content gradually decreases.

The aim of the current study was to explore the dose-dependent effects of formula protein contents on infant body growth during different stages in the first 6 months of life by performing a systematic review and meta-analysis. To achieve our goal, we separated infants into six age groups: 0–1, 1–2, 2–3, 3–4, 4–5, and 5–6 months. We also categorized infant formulas into four groups according to their protein concentrations. Our study highlighted the possible metabolic consequences of excess protein intake during early infancy and provides valuable information to academia and policy-makers.

## 2. Materials and Methods

### 2.1. Literature Screening

The guidelines in the Preferred Reporting Items for Systematic Review and Meta-Analysis Protocols (PRISMA-p) were followed [18]. We conducted a search of the PubMed, Web of Science, and Springer databases. The searching strategy of “((infant formula) NOT (low birth weight) NOT (preterm) NOT (hydrolyzed)) AND ((trial) OR (feeding) OR (composition) OR (content) OR (growth) OR (development) OR (weight) OR (length) OR (height) OR (head circumference) OR (weight gain) OR (length gain) OR (height gain) OR (tolerance) OR (adequacy) OR (safety))” was used. The literature search was completed in August 2021. A total of 2518 duplicates and obviously irrelevant articles were removed after reading the titles and abstracts. The clinical trial articles were screened using the inclusion and exclusion criteria listed in PICOS (Supplementary Table S1). The Cochrane Collaboration’s tool for assessing risk of bias was used while including the following criteria: adequacy of sequence generation; allocation concealment; blinding of participants, personnel, and outcome assessors; incomplete outcome data; selective reporting; and other biases [19]. Notably, studies were not excluded based on these results. Items were scored as having low, high, or unknown risk of bias. The literature screening process was performed independently by two investigators (Q.R. and K.L.). Discrepancies were discussed in the presence of W.Z. until consensuses were reached.

### 2.2. Data Extraction and Analysis

The means, standard deviations (SDs), and a number of samples about growth outcomes of infants during the first six months of life were extracted, which were weight gain, height gain, and BMI. Data were pooled according to age and protein content. We categorized the studied formulas into four groups according to their protein content: <1.8, 1.8–2.0, 2.1–2.2, and >2.2 g/100 kcal. The data were pooled using the meta-analysis approach. Forest plots were generated to compare the growth outcomes of infants fed exclusively with infant formula with different protein/energy ratios with those of infants fed exclusively with breast milk. In the studies that reported continuous variables as means, the mean difference (MD) between the experimental and control groups with 95% confidence intervals (CIs) was calculated. Heterogeneity was assessed using the I^2^ statistic defined by the Cochrane Handbook for Systematic Reviews. A fixed-effects model was used when there was no significant heterogeneity among studies; otherwise, a random-effects model was used. *p* values less than 0.05 were considered statistically significant. Microsoft Excel (Microsoft Corporation, Redmond, WA, USA), Review Manager v5.4 (The Cochrane Collaboration, London, UK), and R v4.1.2 were used for data extraction and analysis. Data extraction and analysis were performed independently by two investigators (Q.R. and K.L.). Discrepancies were discussed in the presence of W.Z. until consensuses were reached.

## 3. Results

**Figure 1:**
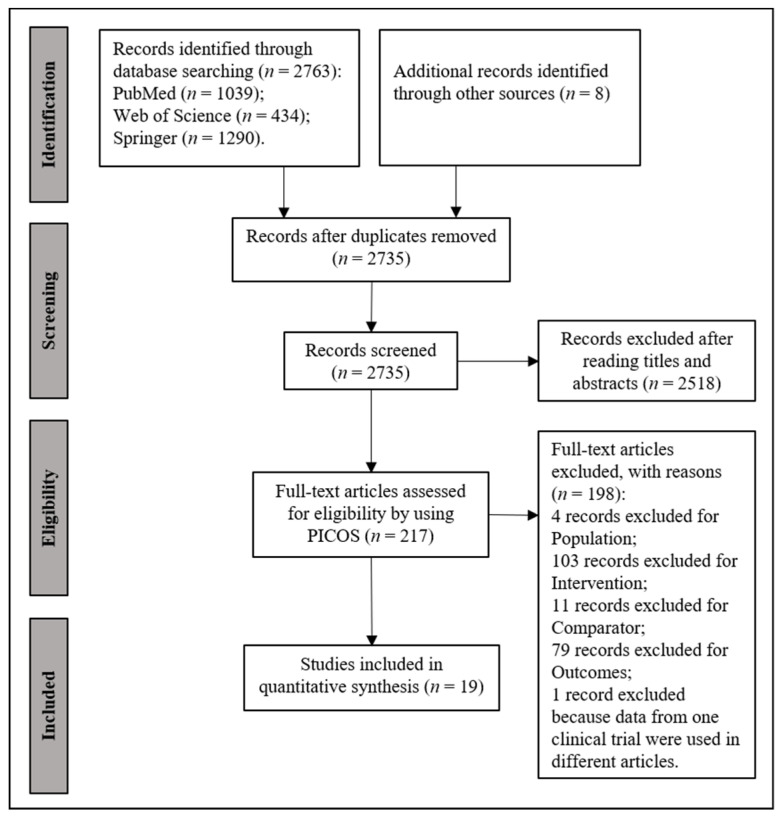
Flow chart of literature searching and screening.

A total of 19 articles were included and assessed in our analysis (Figure 1 and Supplementary Figure S1). Eleven studies were supported by for-profit institutes. The studies were conducted in 17 countries in East Asia, Europe, and North America between 1992 and 2021 [20,21,22,23,24,25,26,27,28,29,30,31,32,33,34,35,36,37,38]. Ages at enrollment ranged from birth to 15 postnatal days; feeding duration ranged from enrollment to 6 months; and protein contents of formulas ranged from 1.4 to 2.7 g/100 kcal (Supplementary Table S2).

**Figure 2:**
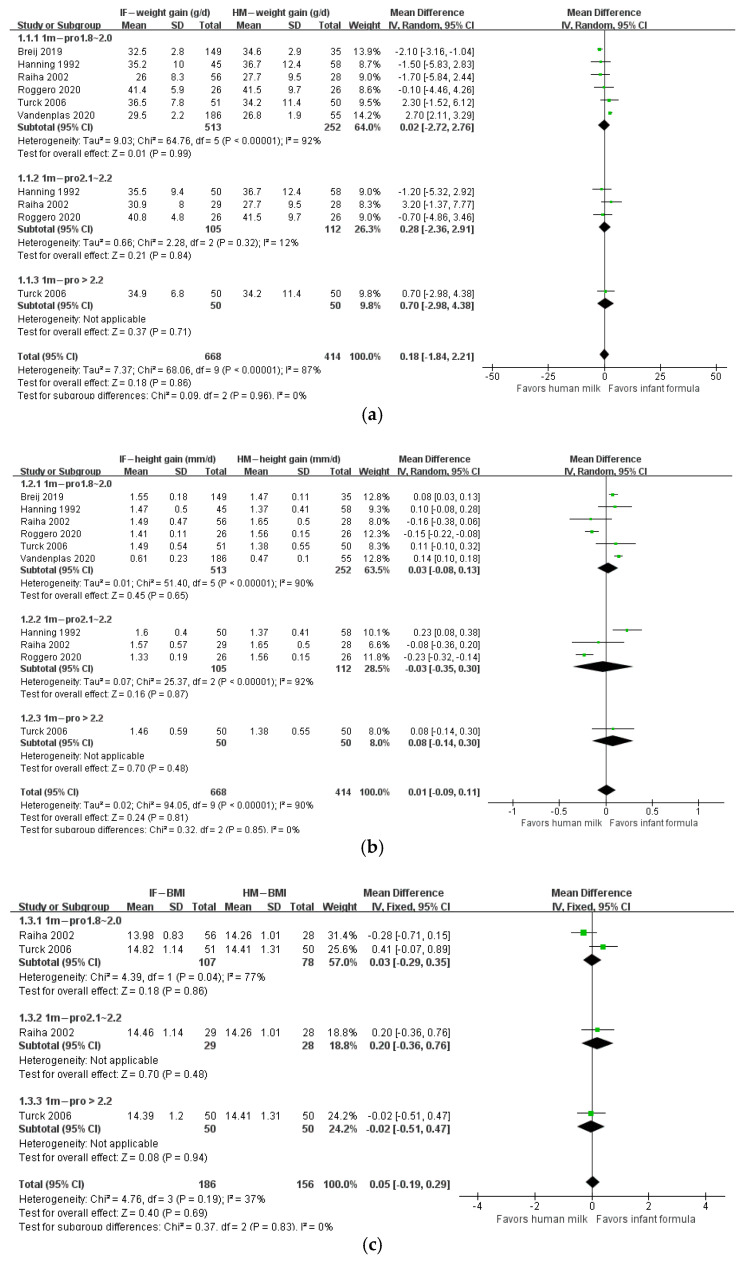
Forest plot of the effects of protein content of infant formula on the weight gain, height gain, and BMI of infants at 1 month of age. (**a**) The effects on weight gain. (**b**) The effects on height gain. (**c**) The effects on BMI.

**Figure 3:**
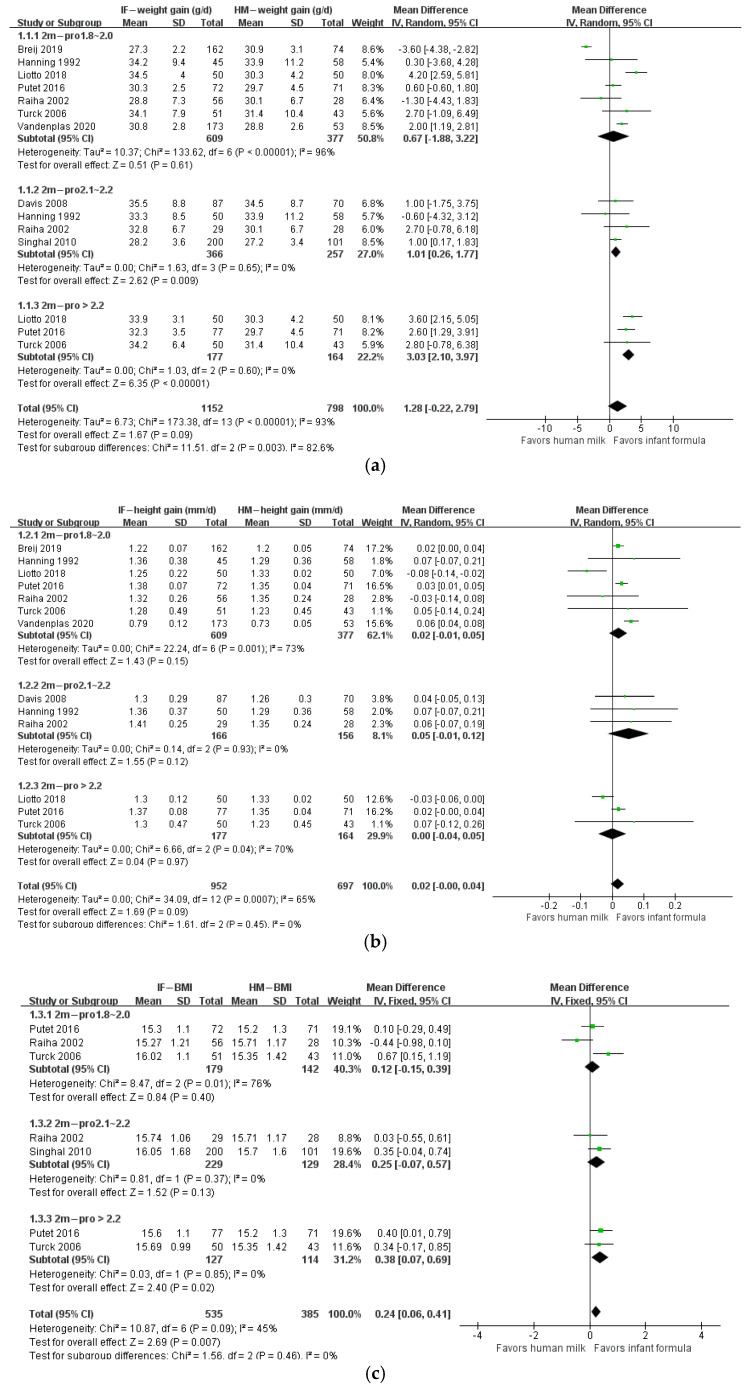
Forest plot of the effects of protein content of infant formula on the weight gain, height gain, and BMI of infants at 2 months of age. (**a**) The effects on weight gain. (**b**) The effects on height gain. (**c**) The effects on BMI.

**Figure 4:**
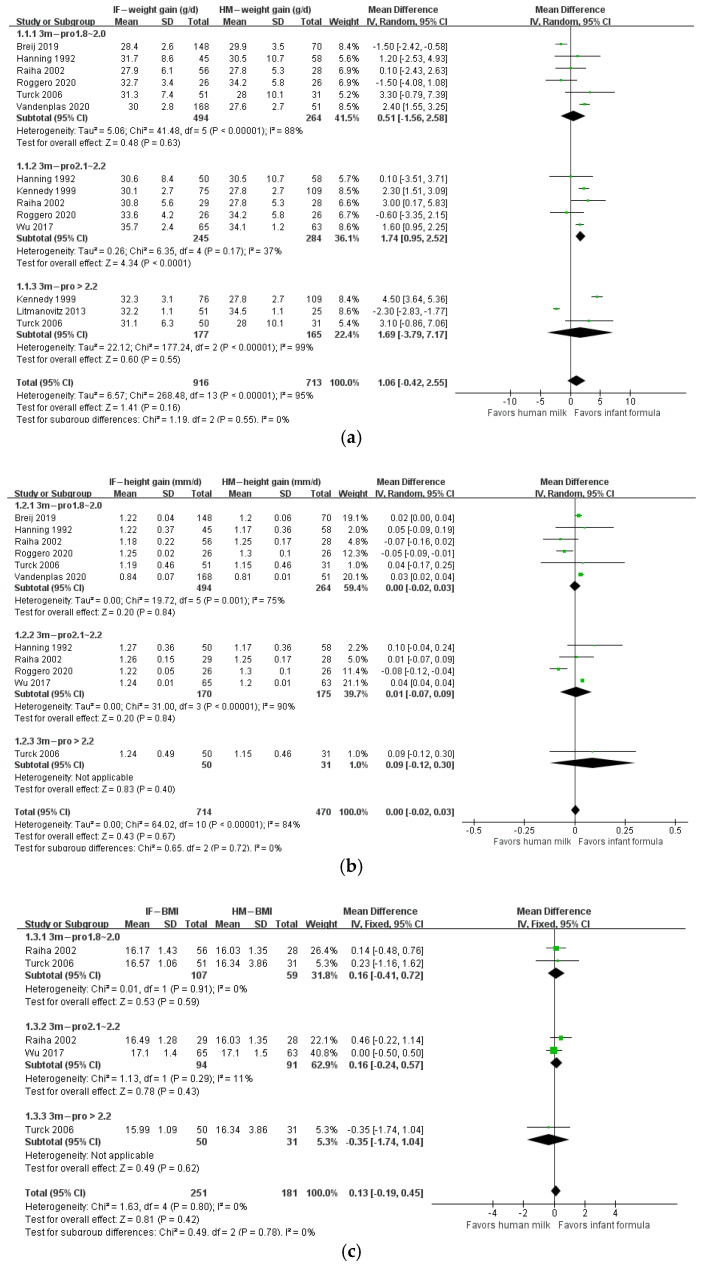
Forest plot of the effects of protein content of infant formula on the weight gain, height gain, and BMI of infants at 3 months of age. (**a**) The effects on weight gain. (**b**) The effects on height gain. (**c**) The effects on BMI.

**Figure 5:**
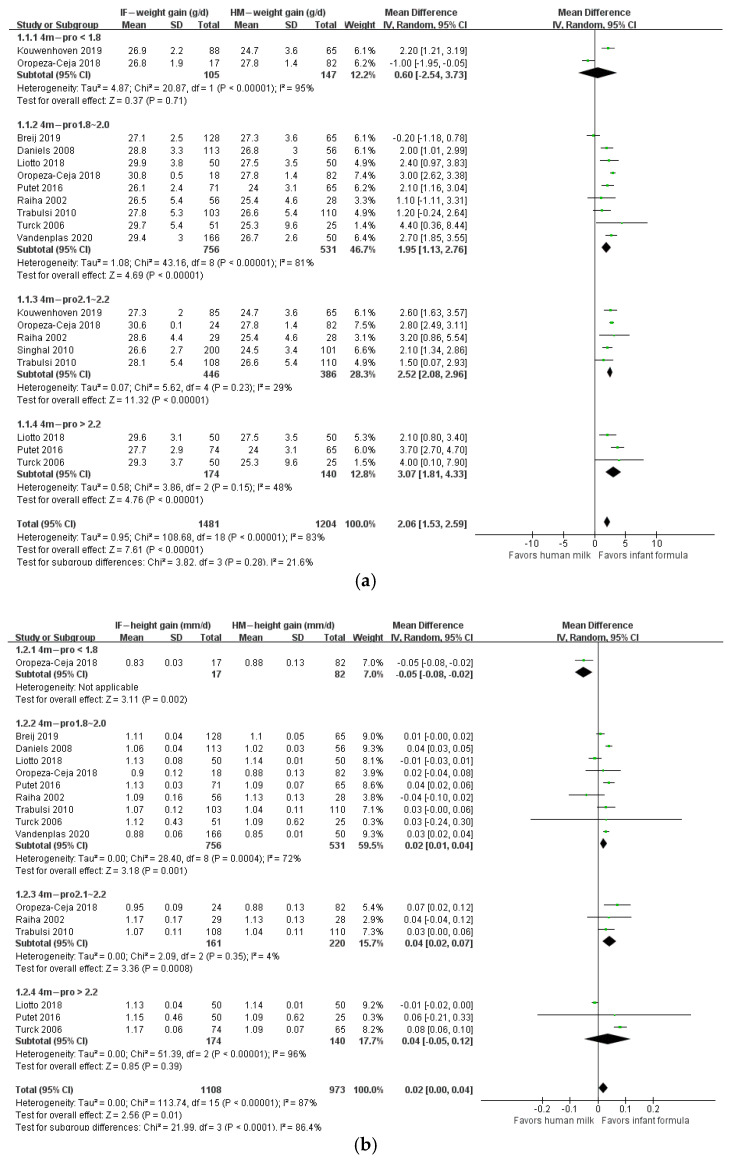

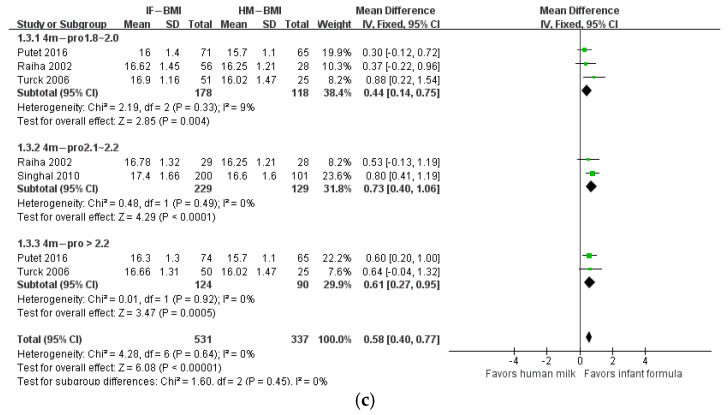
Forest plot of the effects of protein content of infant formula on the weight gain, height gain, and BMI of infants at 4 months of age. (**a**) The effects on weight gain. (**b**) The effects on height gain. (**c**) The effects on BMI.

**Figure 6:**
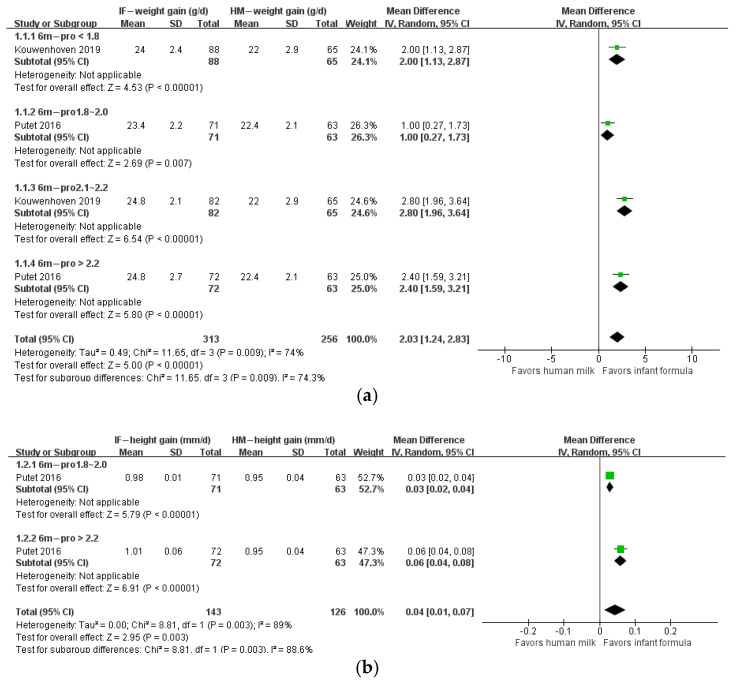

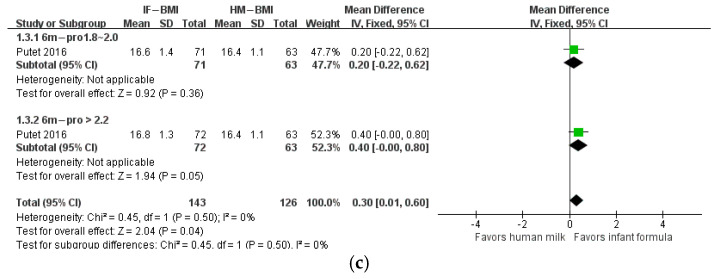
Forest plot of the effects of protein content of infant formula on the weight gain, height gain, and BMI of infants at 6 months of age. (**a**) The effects on weight gain. (**b**) The effects on height gain. (**c**) The effects on BMI.

**Figure 7:**
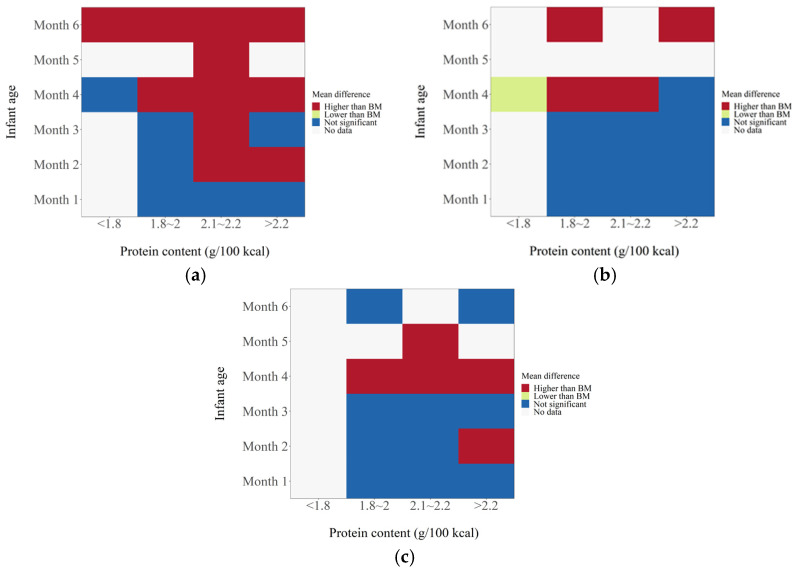
Heat map of the effects of protein content of infant formulas on the growth of infants. (**a**) The effects on weight gain. (**b**) The effects on height gain. (**c**) The effects on BMI.

The protein/energy ratio of infant formula in included studies ranged from 1.4 to 2.7 g/100 kcal. We categorized study formulas into four groups according to their protein content: <1.8, 1.8–2.0, 2.1–2.2, and >2.2 g/100 kcal. At 1 month of age, body weight gain, height gain, and BMI in formula-fed infants were not significantly different from those in breastfed infants (Figure 2). At 2 months of age, body weight gain in infants who consumed formula with protein content 2.1–2.2 g/100 kcal (*n* = 623; MD: 1.01 g; 95% CI: 0.26, 1.77) or higher than 2.2 g/100 kcal (*n* = 341; MD: 3.03 g; 95% CI: 2.10, 3.97) was significantly higher than that in breastfed infants, and BMI in infants that consumed formula with protein content higher than 2.2 g/100 kcal (*n* = 241; MD: 0.38 kg/m^2^; 95% CI: 0.07, 0.69) was significantly higher than that in breastfed infants, whereas height gain in formula-fed infants was not different from that in breastfed infants (Figure 3). At 3 months of age, body weight gain in infants in the 2.1–2.2 g/100 kcal group was higher than that in their breastfed counterparts (*n* = 529; MD: 1.74 g; 95% CI: 0.95, 2.52), whereas height gain and BMI were not different between formula- and breastfed infants (Figure 4). At 4 months of age, body weight gain (*n* = 2685; MD: 2.06 g; 95% CI: 1.53, 2.59), height gain (*n* = 2081; MD: 0.02 mm; 95% CI: 0.00, 0.04), and BMI (*n* = 868; MD: 0.58 kg/m^2^; 95% CI: 0.40, 0.77) in formula-fed infants were significantly higher than those in breastfed infants in all formulas, except that weight gain in infants who consumed formula with protein content lower than 1.8 g/100 kcal was not significantly different from that in breastfed infants (Figure 5). Beyond 4 months of age, body weight gain and height gain in formula-fed infants were significantly higher than those in breastfed infants in all formulas (Figure 6). No forest plot was created of the effects of protein content of infant formula on the weight gain and height gain of infants at 5 months of age, because there was only one study on the weight gain of infants at 5 months of age. In that study, the protein level of 2.2 g/100 kcal was investigated. Body weight gain and BMI in the formula-fed infants were higher than those in breastfed infants. A heat map of the effects of protein content of infant formula on the growth of infants is depicted in Figure 7. It summarizes the differences between breastfeeding and formula feeding in terms of weight gain, height gain, and BMI. 

## 4. Discussion

Our study analyzed the relationship between the protein content of infant formulas and infant growth in a longitudinal manner in the first 6 months of life. We found that, in the first month of life, growth was not different between formula- and breastfed infants. During 2–3 months of life, growth was faster in infants who consumed formulas with protein content higher than 2.0 g/100 kcal. After 3 months, formula-fed infants grew faster than breastfed infants. To our best knowledge, our study was the first systematic review and meta-analysis that investigated the association between the protein content of infant formulas and infant growth in the first 6 months of life. 

We included a total of 19 papers in our study by applying the inclusion and exclusion criteria in the PICOS table (Supplementary Table S1). Some studies that were included by others were not included by us because they did not meet our standards. For example, studies by P.M. Akeson et al. did not meet our standards because the subjects were not exclusively breastfed or exclusive formula fed [39,40]. Another example is that a study by Weber et al. was not included because the study did not identify anthropometric assessments of infants during the first 6 months of life [41]. 

The difference in protein concentrations between human milk and infant formulas may explain the faster growth trajectory in formula-fed infants than in breastfed infants. Human milk protein concentrations decrease gradually from 2.13 to 1.14 g/dL in the first 90 days of lactation and remain stable thereafter [42]. By contrast, formula-fed infants, in the first 6 months of life, consume the same formulas, which typically range from 1.4 to 1.5 g of protein/dL. Accordingly, infant formulas may provide 30% more protein than human milk after the infant is 2 months old [42]. Because the digestibility, amino acid profile, and protein efficiency of human milk protein are superior to those of other protein sources such as bovine milk, it is rational for infant formula to provide higher concentrations of protein. It is possible that the food calories introduced by excessive protein in infant formula cause the faster growth velocity in formula-fed infants. Studies have also suggested that excessive protein may raise the levels of IGF-1, which may mediate faster weight gain. 

The findings of our work agreed with other reviews. Patro-Gołąb et al. concluded that different formula protein concentrations did not affect mean weight at 3 months as observed in a meta-analysis of four studies. Lower mean weight and weight z scores obtained from the infants fed lower-protein formulas were observed only from 6 to 12 months of age [17]. Aurore Camier et al. determined whether the variability in the protein content of infant formula used in France over the period 2003–2012 was significantly associated with early growth in children. Their findings confirmed the positive association between infant formula protein content and weight- and BMI-for-age z-scores up to 18 months. Among formula-fed infants, the lowest protein-content group had the lowest anthropometric z-scores, although these scores remained higher than those for breastfed infants [43]. A review by Hornell et al. published in 2013 focused on protein intake in the diet (not only in milk formula) of children of different ages (0 to 18 years) and its relation to health. The authors concluded that higher protein intake during infancy was associated with increased growth, a higher BMI during childhood, and an increased risk of being overweight later in life [44]. Fenton et al. found that low-certainty evidence suggested that higher protein intake (≥3.0 g/kg/d but <4.0 g/kg/d) from formula accelerates weight gain of preterm or low birth weight infants [45]. 

Our study and others described above showed that protein content of infant formula was associated with early growth in children. However, there is insufficient direct evidence to evaluate the effects of reducing protein concentrations in infant formula on long term outcomes related to decreased risk of later obesity. According to the “early protein hypothesis”, however, reducing early protein intake is a potential lever for obesity prevention. Therefore, a relatively lower protein/energy ratio in infant formula may reduce the risk of overweight and obesity in children. In fact, many studies have investigated the safety of infant formulas with reduced protein levels in order to simulate human milk. More notably, when reducing the protein content of infant formula, attention should be paid to the composition of amino acids. Otherwise, unreasonable amino acid patterns may lead to limited protein synthesis during the rapid growth of infants. For this reason, using a low-protein formula with an amino acid composition modified according to the estimated infant requirements has been suggested [46].

This review had some limitations. Because we separated protein contents into four levels and categorized the postnatal life into six stages, the number of studies in each category was limited. For example, at the age of under 1 month, there was only one study in each of the protein levels of <1.8, 1.8–2.0, 2.1–2.2, and >2.2 g/100 kcal. The limited number of studies diminished the power of the meta-analysis. Nevertheless, our results highlighted the dose-dependent effects of formula protein content on infant growth and the effects of formula feeding on growth in different postnatal ages, which could encourage future investigations by using animal models, human studies, and systematic reviews and meta-analysis. Additionally, we only analyzed the infants’ growth during first six months of the life, because nutrient intake during this time is provided only by breast or formula. Other sources of nutrition in the diet also play an important role after the introduction of complementary feeding. This review included studies of exclusive breastfeeding and exclusive formula feeding patterns, but mixed milk feeding (MMF) was not considered. MMF defined as the combination of breastfeeding and formula feeding during the same period in term infants [47]. There may be some changes in the growth performance of differently fed infants. Feng et al. conducted a longitudinal study of Chinese breastfeeding infant growth and development. It showed that infants who were nonexclusively breastfed had lower weights than those who were exclusively breastfed but that the difference was not statistically significant. However, there was an interaction between feeding patterns and age. The average weight of babies who were nonexclusively breastfed increased faster than those who were exclusively breastfed with age [48]. MMF is a widespread feeding reality. It is very important to evaluate MMF’s influence on infants’ nutritional status, growth, development, and health status in the short and long terms. Energy deposition as a percentage of total energy requirements decreases from 40% at age 1 month to 1–2% from 12 months until midadolescence. Therefore, weight gain during infancy is more closely related to energy intake than is weight gain in childhood or in later life [49]. Energy intake in early infancy is mainly from breast milk or formula, so breast milk or formula intake is closely related to weight gain during infancy. Eight of the twenty articles included in this systematic review reported infant formula intake (Supplementary Table S2). The results showed no difference between groups, except that the studies of Turck and Putet showed higher intakes of infants in the high-protein powder group [22,24]. Because of the limited evidence, it was unclear whether protein content affected infant formula intake. Studies have shown that breast milk or formula intake is associated with infant appetite traits. Appetitive traits are thought to be largely innate and stable. Infant appetitive profiles characterized by high food responsiveness and low satiety responsiveness are prospectively associated with more rapid weight gain. However, parental attitudes to following healthy infant feeding guidelines attenuate the effects of infant appetitive traits on infant milk intake and body weight [11].

Human milk is the most effective and natural food for infants. Therefore, many ingredients are being taken to create an infant formula mimicking the composition of the human milk as closely as possible for infants who cannot be breastfed. The growth outcomes of infants fed formula supplemented with these ingredients are worth evaluating. Some systematic reviews about comparisons between infants fed with standard formula and those fed with milk fat globule membrane-supplemented formulas, palm olein-containing formulas, prebiotic-supplemented formulas, or taurine-containing formulas revealed no differences in anthropometric measurements [50,51,52,53]. A meta-analysis by Wang et al. indicated that nucleotide supplementation could increase the rate of weight gain; however, it could not conclude that it affected other anthropometric measurements [54]. A meta-analysis by Sun et al. showed that there was lower weight gain in the probiotics group than in the control group but that this difference was not statistically significant [55]. Therefore, even if the relevant components of infant formula milk powder underwent the aforementioned changes, the impact on infants’ weight and other relevant anthropometric parameters would be very small.

## 5. Conclusions

Our meta-analysis indicated that exclusively formula-fed infants would have visibly higher weight gain starting from four months of age. The growth outcomes of infants fed with infant formula with a relatively low protein/energy ratio, compared with those of infants fed with infant formula with a relatively high protein/energy ratio, were close to those of breastfed infants. To our knowledge, we collated the largest number of studies on the effects of breastfeeding and feeding with formulas with different protein/energy ratios on the growth outcomes of infants during the first six months the life. The findings of this review were similar to those of other reviews addressing the effect of the intake of low-protein formula by infants on growth. In addition, the protein content of infant formula is not the only factor that may affect infant growth. Therefore, we also discussed the influence of feeding patterns, formula intake, ingredients of infant formula, and other factors on infant growth. However, we analyzed the infants’ growth only during the first six months of life. There is insufficient direct evidence to evaluate the effects of reducing protein concentrations in infant formula on long-term outcomes related to decreased risk of later obesity. In view of the limited available evidence, more studies demonstrating effects on long-term health outcomes are needed.

## Data Availability

Not applicable.

## Supplementary Materials

The following supporting information can be downloaded at: https://www.mdpi.com/article/10.3390/nu14112255/s1, Figure S1: Risk of bias in the included studies; Table S1: Inclusion and exclusion criteria for selecting articles (PICOS); Table S2: Characteristics of included studies.
