# Supplementary material for: The Association of Formula Protein Content and Growth in Early Infancy: A Systematic Review and Meta-Analysis
Source: Nutrients. 2022 May 28;14(11):2255. doi: 10.3390/nu14112255 (PMC9183142; doi:10.3390/nu14112255)
Supplement: Supplementary file 1 [file nutrients-14-02255-s001.zip › nutrients-1713384-supplementary.pdf]

Title: The Association of Formula Protein Content and Growth in Early Infancy: A Systematic Review and Meta-Analysis

supplementary materials

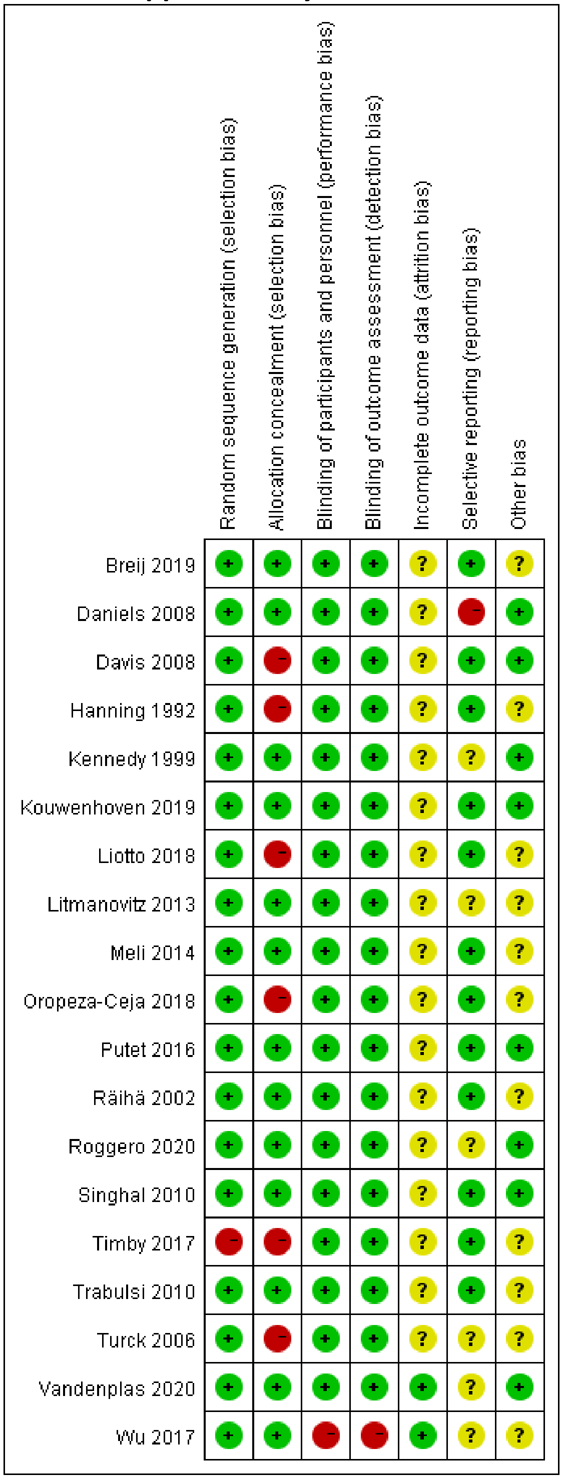

Figure S1. Risk of bias in the included studies.

Table S1. Inclusion and exclusion criteria for selecting articles (PICOS)

Parameter	Inclusion criteria	Exclusion criteria
**Population**	Healthy infants; term infants Healthy term infants aged <6 months, receiving exclusive breastfeeding or exclusive formula feeding.	Non-human; mothers or infants with defined diseases or disorders; premature infants; Low birth weight infants.
**Intervention**	Milk-based infant formula; Infant formula with a protein/energy ratio	None-milk-based infant formula; Preterm infant formula; Partially hydrolyzed formula; Extensively hydrolyzed formula; Amino acid formula; undefined protein/energy ratio
**Comparator**	Breastfeeding infants	N/A
**Outcomes**	Anthropometric assessments (weight, length, BMI, weight gain, length gain) in the first 6 months of infant's life; data were expressed as means or medians	Infant anthropometric assessments (weight, length, BMI, weight gain, length gain) in the first 6 months of infant's life not specified.
**Study design**	A controlled and randomized clinical trial	Review articles; abstracts; articles without access to full-text

Table S2. Characteristics of included studies

Reference	Study type	Setting	Study population	Group and Number of infants (males)	Duration of exposure	Anthropometric Assessments	Funding
Davis 2008	A prospective, multicenter, masked, randomized clinical trial	N.S.	Healthy term infants, postnatal age ≤ 14 days	Group 1: Breast milk, n=88 (39); Group 2: Infant formula (energy: 67 kcal/100 ml; protein: 2.2 g/100 kcal; carbohydrate: 10.9 g/100 kcal; fat: 5.4 g/100 kcal), n=64 (30); Group 3: Infant formula (energy: 67 kcal/100 ml; protein: 2.1 g/100 kcal; carbohydrate: 10.9 g/100 kcal; fat: 5.4 g/100 kcal), n=64 (29)	8 weeks	Weight, length, head circumference, weight gain, length gain, head circumference gain, the daily volume of formula intake	Wyeth Nutrition (Collegeville, PA, USA)
Singhal 2010	A randomized controlled blind trial	Leicester and Nottingham, United Kingdom	Healthy term infants, at birth	Group 1: Breast milk, n=101 (51); Group 2: Infant formula (energy: 68 kcal/100 ml; protein: 2.2 g/100 kcal; carbohydrate: 10.3 g/100 kcal; fat: 5.6 g/100 kcal), n=200 (117)	20 weeks	Weight, length, head circumference, Weight-for-age Z-score, Height-for-age Z-score, Head circumference for age Z-score, body mass index (BMI), the daily volume of formula intake	The Medical Research Council with a charitable contribution from the H. J. Heinz Company Ltd (Hayes, Middlesex, United Kingdom)
Turck 2006	A controlled, prospective, randomized and double-blind feeding trial	Lille, Marseilles, Paris and Reims	Healthy term infants, postnatal age < 7 days	Group 1: Breast milk, n=55 (28); Group 2: Infant formula (energy: 67 kcal/100 ml; protein: 1.8 g/100 kcal; carbohydrate: 11.2 g/100 kcal; fat: 5.4 g/100 kcal), n=51 (27); Group 3: Infant formula (energy: 67 kcal/100 ml; protein: 2.6 g/100 kcal; carbohydrate: 11.6 g/100 kcal; fat: 4.5 g/100 kcal), n=50 (28)	120 (±4) days	Weight, length, head circumference, weight gain, length gain, head circumference gain, daily gains in weight, length and head circumference, body mass index (BMI), the daily volume of formula intake	Not reported
Meli 2014	A randomized, double-blind, single-center trial	Palermo, Italy	Healthy term infants, postnatal age≤14 days	Group 1: Breast milk, n=30 (14); Group 2: Infant formula (energy: 67 kcal/100 ml; protein: 1.8 g/100 kcal), n=281 (161)	4 months	Weight, length, head circumference, weight gain, length gain, head circumference gain	Nestlé Nutrition
Putet 2016	A randomized, double-blind, parallel group, controlled, single-centre study	Lyon, France,	Healthy term infants, postnatal age < 7 days	Group 1: Breast milk, n=84 (42); Group 2: Infant formula (energy: 68 kcal/100 ml; protein: 1.8 g/100 kcal), n=74 (41); Group 3: Infant formula (energy: 68 kcal/100 ml; protein: 2.7 g/100 kcal) n=80 (42)	12 months	Weight, length, head circumference, body mass index (BMI), the daily volume of formula intake	Nestlé, Switzerland
Litmanovitz 2013	A randomized, double-blind controlled, longitudinal trial	Israel	Healthy term infants, postnatal age < 14 days	Group 1: Breast milk, n=25 (15); Group 2: Infant formula (protein: 2.4 g/100 kcal), n=51 (25)	12 weeks	Weight, length	Not reported
Trabulsi 2010	A randomized, controlled, double-blind study	N.S.	Healthy term infants, postnatal age 5-14 days	Group 1: Breast milk, n=112; Group 2: Infant formula (energy: 67 kcal/100 ml; protein: 2.1 g/100 kcal; carbohydrate: 10.8 g/100 kcal; fat: 5.4 g/100 kcal), n=112; Group 3: Infant formula (energy: 67 kcal/100 ml; protein: 1.9 g/100 kcal; carbohydrate: 10.8 g/100 kcal; fat: 5.4 g/100 kcal), n=112	4 months	Weight, length, weight gain, length gain, head circumference gain	Wyeth Nutrition, Ireland

Kennedy 1999	A randomized, double-blind trial	United Kingdom	Healthy term infants, newborns	Group 1: Breast milk, n=120 (65);<br>Group 2: Infant formula (energy: 70 kcal/100 ml; protein: 2.3 g/100 kcal; carbohydrate: 10.1 g/100 kcal; fat: 5.6 g/100 kcal), n=103 (65);<br>Group 3: Infant formula (energy: 73 kcal/100 ml; protein: 2.2 g/100 kcal; carbohydrate: 9.7 g/100 kcal; fat: 5.8 g/100 kcal), n=100 (54)	12 weeks	Weight, length, head circumference	Not reported
Breij 2019	A randomized, double-blind, controlled, prospective, multicountry, (growth) equivalence trial	Netherlands, Belgium, France, Singapore	Healthy term infants, postnatal age ≤ 35 days	Group 1: Breast milk, n=77 (38);<br>Group 2: Infant formula (energy: 66 kcal/100 ml; protein: 2 g/100 kcal; carbohydrate: 11.1 g/100 kcal; fat: 5.2 g/100 kcal), n=174 (84)	17 weeks	Weight, length, head circumference	Not reported
Liotto 2018	A prospective, controlled, single-blinded randomized trial	N.S.	Healthy term infants, postnatal age < 3 weeks	Group 1: Breast milk, n=50;<br>Group 2: Infant formula (energy: 65 kcal/100 ml; protein: 1.9 g/100 kcal; carbohydrate: 12.3 g/100 kcal; fat: 4.8 g/100 kcal), n=50;<br>Group 3: Infant formula (energy: 68 kcal/100 ml; protein: 2.5 g/100 kcal; carbohydrate: 10.4 g/100 kcal; fat: 5.1 g/100 kcal), n=50	4 months	Weight, length, head circumference, Weight-for-age Z-score, Height-for-age Z-score, Head circumference for age Z-score, the daily volume of formula intake	Humana Italia s.p.a
Oropeza-Ceja 2018	A randomized, single-blind controlled, longitudinal trial	Queretaro, Mexico	Healthy term infants, postnatal age≤40 days	Group 1: Breast milk, n=82;<br>Group 2: Infant formula (energy: 65 kcal/100 ml; protein: 1.4 g/100 kcal), n=17;<br>Group 3: Infant formula (energy: 68 kcal/100 ml; protein: 1.9 g/100 kcal), n=18;<br>Group 4: Infant formula (energy: 70 kcal/100 ml; protein: 2.2 g/100 kcal), n=24	4 months	Weight, length, head circumference, Weight-for-age Z-score	Consejo Nacional de Ciencia y Tecnología: 199586
Daniels 2008	A prospective, randomized, and controlled, blinded design with 3 formula-fed groups in parallel	Adelaide, South Australia	Healthy term infants, postnatal age 3-5 days	Group 1: Breast milk, n=56 (27);<br>Group 2: Infant formula (energy: 67 kcal/100 ml; protein: 1.8 g/100 kcal), n=113 (52)	16 weeks	Weight, length, head circumference	Nestlé
Räihä 2002	A controlled, blind, parallel, and prospective feeding study	di Palermo, Palermo, Macedonio Melloni, Milan	Healthy term infants, postnatal age < 28 days	Group 1: Breast milk, n=28;<br>Group 2: Infant formula (protein: 2.2 g/100 kcal), n=29;<br>Group 3: Infant formula (protein: 1.8 g/100 kcal), n=56	120 days	Weight, length, weight gain, length gain, body mass index (BMI), the daily volume of formula intake	Nestlé Ltd
Timby 2017	A prospective double-blinded randomized controlled trial	N.S.	Healthy term infants, postnatal age < 2 months	Group 1: Breast milk, n=73 (29);<br>Group 2: Infant formula (energy: 66 kcal/100 ml; protein: 1.9 g/100 kcal), n=73 (41);<br>Group 3: Infant formula (energy: 60 kcal/100 ml; protein: 2 g/100 kcal), n=76 (40)	12 months	Weight, length	VaÈsterbotten County Council, Sweden (TUA, ALF), Sweden's Innovation Agency (Vinnova), and Semper AB, Sweden
Roggero 2020	A monocentric, randomised, double-blind, placebo-	Milan	Healthy term infants, postnatal age<7 days	Group 1: Breast milk, n=26;<br>Group 2: Infant formula (energy: 69 kcal/100 ml; protein: 2 g/100 kcal; carbohydrate: 10.7 g/100 kcal; fat: 5.2 g/100 kcal), n=26;	3 months	Weight, length, head circumference	Heinz Italia s.p.a

	controlled, parallel group trial			Group 3: Infant formula (energy: 68 kcal/100 ml; protein: 2.1 g/100 kcal; carbohydrate: 10.7 g/100 kcal; fat: 5.3 g/100 kcal), n=26;			
Hanning 1992	A randomized, controlled trial	Hamilton, Ontario	Healthy term infants, newborns	Group 1: Breast milk, n=58;<br>Group 2: Infant formula (protein: 2.2 g/100 kcal), n=50;<br>Group 3: Infant formula (protein: 2 g/100 kcal), n=45	12 weeks	Weight, weight gain, length gain, head circumference gain, the daily volume of formula intake	Wyeth-Ayerst International Limited.
Wu 2017	A randomized, controlled, parallel, multicenter, clinical trial	China	Healthy term infants, postnatal age≤14 days	Group 1: Breast milk, n=63 (29);<br>Group 2: Infant formula (energy: 68 kcal/100 ml; protein: 2.2 g/100 kcal; carbohydrate: 10.3 g/100 kcal; fat: 5.5 g/100 kcal), n= 65 (29)	13 weeks	Weight, length, head circumference, body mass index (BMI), Weight-for-age Z-score	Twelfth Five Year National Science and Technology Plan Project (grant number 2013BAD18B03)
Kouwenhoven 2019	A double-blind, randomized, controlled, equivalence trial	Netherlands, Germany	Healthy term infants, postnatal age≤45 days	Group 1: Breast milk, n=67 (31);<br>Group 2: Infant formula (energy: 67 kcal/100 ml; protein: 1.7 g/100 kcal; carbohydrate: 11.5 g/100 kcal; fat: 5.1 g/100 kcal), n=90 (41);<br>Group 3: Infant formula (energy: 67 kcal/100 ml; protein: 2.1 g/100 kcal; carbohydrate: 11 g/100 kcal; fat: 5.1 g/100 kcal), n=88 (41);	6 months	Weight, length, head circumference	European Union's Seventh Framework Programme (FP7/2007–2013), project Early Nutrition under grant agreement no. 289346 and the European Research Council Advanced Grant META-GROWTH ERC-2012-AdG–no.322605.
Vandenplas 2020	A double-Blind, randomized, controlled, multi-country trial	Belgium, Hungary, Poland, Spain, Ukraine,	Healthy term infants, postnatal age≤14 days	Group 1: Breast milk, n=58 (27);<br>Group 2: Infant formula (energy: 66 kcal/100 ml; protein: 2 g/100 kcal; carbohydrate: 11.1 g/100 kcal; fat: 5.2 g/100 kcal), n=196 (93);	17 weeks	Weight, length, head circumference, weight gain, length gain, head circumference gain, the daily volume of formula intake	Danone Nutricia Research
